# Heavy Cannabis Use Associated with Wernicke’s Encephalopathy

**DOI:** 10.7759/cureus.5109

**Published:** 2019-07-09

**Authors:** Amit Chaudhari, Zi Ying Li, Alan Long, Arash Afshinnik

**Affiliations:** 1 Medicine, University of California, San Francisco-Fresno, Fresno, USA; 2 Internal Medicine, University of California, San Francisco-Fresno, Fresno, USA

**Keywords:** cannabis, cannabis hyperemesis syndrome, seizures, status epilepticus, hyponatremia, wernicke’s encephalopathy, cannabis side effects, thiamine deficiency, vitamin b1

## Abstract

Cannabis use accounts for more than 149,000 hospital visits annually. As more states legalize recreational Cannabis, side effects that are currently rare or unknown will become increasingly more common. Here, we present one such rare case of Cannabis-induced hyperemesis causing Wernicke’s encephalopathy.

This is an investigational case report utilizing retrospective data from electronic medical records.

A 41-year-old patient presented to the hospital in status epilepticus secondary to severe vomiting and hyponatremia. He was given one dose of thiamine, glucose and folate and admitted to the medical ICU. His history was significant for remote alcohol use (1-2 beers/week about 20 years ago) and heavy marijuana use from strains grown in the patient’s own backyard. A diagnosis of Cannabis Hyperemesis Syndrome was made. Seizures resolved after correction of electrolytes, and he became awake and alert with no focal deficits. His neurological exam after he was clinically stable showed memory deficits including confabulations (e.g., incorrectly listing occupation) and delusions (e.g., praying to a queen bee). An extensive workup including routine laboratory testing, infectious panels, and autoimmune studies was entirely negative. On the day of admission, brain magnetic resonance imaging (MRI) was performed showing bilateral thalamic hyperintensities on T2 FLAIR MRI. Wernicke's encephalopathy (WE) remained most likely and intravenous thiamine led to a gradual improvement in the patient’s symptoms. He is now two months into rehabilitation and continues to make progress in recalling life events.

Alcohol abuse is empirically treated with thiamine whereas Cannabis, unlike alcohol, is presumed to induce hyperphagia and nutritional supplements are often not initiated. However, foods ingested by Cannabis users are nutritionally deficient due to underline malabsorption. In addition, Cannabis-induced vomiting can further cause malnutrition. Complications, like Wernicke’s encephalopathy, can be prevented by supplementing thiamine early in Cannabis intoxication.

## Introduction

Cannabis is now the most widely used illicit drug in the United States, with more than 11 million young adults ages 18 to 25 having used marijuana in the past year [1,2]. Its two active ingredients - psychoactive delta-9-tetrahydrocannabinol (THC) and non-psychoactive cannabidiol (CBD) - cause transient euphoric reactions including relaxation, feelings of well-being, and increased sociability. Side effects have traditionally been harder to quantify as cannabis users often simultaneously indulge in other substances, but case reports of anxiety, paranoia, gastrointestinal symptoms, asthma exacerbations, bronchitis, decreased cognitive functioning, and irreversible brain damage have been reported in literature. While infrequent users experience increased appetite and hyperphagia, long-term cannabis use has been associated with nausea, poor nutritional intake, abdominal pain and hyperemesis.

Cannabinoid hyperemesis syndrome (CHS) is estimated to affect 32.9% of marijuana users between the ages of 18 and 49 years, or approximately 2.5 million Americans annually. While the pathophysiology of CHS is still under investigation, hyperemesis combined with poor nutritional intake and gastrointestinal disturbances has been linked to fluid imbalances, electrolyte disturbances and vitamin deficiencies including thiamine [3-5]. However, advances in scientific understanding have yet to reach clinical settings where cannabis side effects are under-recognized and empiric treatments are not yet advocated. Here, we present a patient who was taken off vitamin supplementation after the diagnosis was refined from general substance abuse including alcoholism to cannabinoid hyperemesis syndrome, which soon led to the development of Wernicke’s encephalopathy (WE) with ongoing neurological deficits.

## Case presentation

A 41-year-old Caucasian male with no significant past medical history was brought in by emergency medical services (EMS) with altered mental status. Per family, he was a high-functioning individual with military service as a Navy nuclear engineer and a subsequent engineering career in the private sector. His last use of alcohol or tobacco was >20 years ago, but family noted an ongoing history of heavy marijuana abuse. He was last seen well about three weeks prior to presentation at a family gathering where he was able to hold conversations and engage in all activities of daily living without any cognitive deficits. Today, he was found unresponsive in the bathroom with a emesis basin by his side. Vital signs on initial examination at the emergency room were notable for a blood pressure of 117/86 mmHg, heart rate of 99, respiratory rate of 19, temperature of 98.6 F, and BMI of 34.5. Neurological exam was significant for a Glasgow Coma Scale (GCS) 10 (eyes to voice, incomprehensible sounds, and localized withdrawal to pain), equal and reactive pupils with nonsustained torsional nystagmus, intact cranial nerves, spontaneous movement of all extremities with no focal deficits. The remainder of the physical exam in the emergency room was unremarkable. Blood cultures were sent and patient received one dose of empiric 250 mg IV thiamine, D50 and folic acid for prevention of possible alcohol-induced encephalopathy.

**Figure 1:**
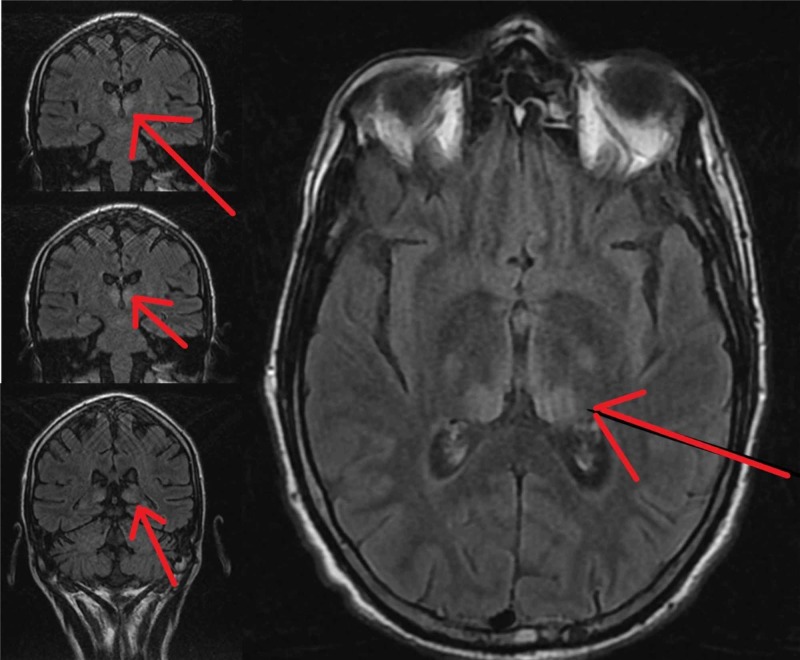
T2 FLAIR MRI of the brain On the T2 FLAIR MRI of the brain it showed bilateral thalamic hyperdensities (red arrows).

On hospital day 2, he remained disoriented and demonstrated periods of unresponsiveness. Lab work was significant only for hemoglobin of 21.4 g/dl, platelet count of 79 k per microliter, sodium level of 125 mEq/L, potassium level of 2.6 mEq/L, hypochloremic metabolic alkalosis, and marijuana on urine toxicology (full lab work results in Table 1). Chest X-ray and head CT were negative. Electroencephalogram (EEG) showed ongoing seizure activity, and he was transferred to the medical ICU for management of status epilepticus. The diagnosis was then refined from general substance abuse including alcoholism to cannabinoid hyperemesis syndrome with hyponatremia-associated seizures. Repletion of electrolytes and starting fosphenytoin and lacosamide led to a cessation of his seizures. However, he continued to be disoriented and failed to show any meaningful clinical improvement in cognition even by hospital day 6. In addition, his verbal responses to historical questions now showed evidence of long-term memory deficits (e.g., inability to recall place of birth), confabulations (e.g., changing occupations and accomplishments), and delusions (e.g., paying homage to a queen bee). An extensive interim workup (Figure 1) was unremarkable except for bilateral thalamic hyperdensities on T2 FLAIR MRI. He was then treated with thiamine 500 mg IV for four days and then 100 mg IV daily for presumed Wernicke’s encephalopathy. Treatment with thiamine gradually improved his GCS to 15 and he became oriented to self and place. On hospital day 18, he was discharged to acute rehab on thiamine and phenytoin where he continues to have intermittent delusions and confabulations and requires ongoing assistance in performing activities of daily living. He is now able to identify family members and recall some memories, as well as demonstrate consistent improvements in cognitive function.

**Table 1:** Full laboratory result

Presenting labs	Results
White blood count (WBC)	7 x 10^9^ per liter
Hemoglobin	21.4 g/dl
Platelet	128 per microliter
Mean corpuscular volume (MCV)	79 fL
Sodium (Na)	125 mEqL
Potassium (K)	2.6 mEqL
Chloride (Cl)	73 mEqL
Bicarbonate (CO2)	32 mEqL
Blood urea nitrogen (BUN)	28 mg/dL
Creatinine (Cr)	1.04 mg/dL
Albumin	4.5 g/dl
Total bilirubin (T. Bili)	2.7 mg/dl
Direct bilirubin (D. Bili)	0.5 mg/dl
Alkaline phosphatase (ALP)	59 U/L
Alanine transaminase (ALT)	45U/L
Aspartate transaminase (AST)	26 U/L
Magnesium (Mg)	1.8 mEqL
Pre albumin	8 g/dl
International Normalized Ratio (INR)	1.1
Partial thromboplastin time (PTT)	31 sec
Lactate dehydrogenase (LDH)	118 U/L
Additional lab	Result
Acetaminophen level	Negative
Salicylate level	Negative
Urine Ethanol	Negative
Urine Amphetamine	Negative
Urine Barbiturates	Negative
Urine Benzodiazepine	Negative
Urine Cannabinoid	POSITIVE
Urine Cocaine	Negative
Urine Opiates	Negative
Urine Oxycodone	Negative
Urine Phencyclidine	Negative
Rapid plasma reagin (RPR)	Negative
Human Immunodeficiency Virus (HIV) antibody and antigen	Negative
Vitamin B1	<7 nmol/L
Folate	<2.5 ng/ml
Vitamin B12	745 pg/ml
Hepatitis C Virus (HCV) Antibody and antigen	Negative
Streptococcus Pneumoniae Urine antigen	Negative
West Nile Virus antibody	<0.90
Herpes Simplex Virus (HSV) 1 & 2	Negative
Anti-N-methyl-D-aspartate antibody (NMDA-Ab)	Negative
Heparin-induced Thrombocytopenia antibody (HIT-Ab)	Negative

## Discussion

Wernicke’s encephalopathy is a devastating neurological disorder caused by thiamine (B1) deficiency. WE is about 900 times more frequent in alcoholics, but has also been described in the setting of malignant disorders, gastrointestinal disease and surgery, hyperemesis gravidarum and other conditions that lead to severe malnutrition [6]. The classic clinical triad of oculomotor dysfunction, cerebellar ataxia and memory loss are often not readily evident, and most patients are diagnosed based solely on history, alterations in mental status, and exclusion of all other etiologies. Though not necessary for diagnosis, T2 FLAIR MRI showing hyperintensities in bilateral thalami, mammillary bodies or medulla can be highly suggestive of WE [7].

In the case above, our patient presented with altered mental status and was appropriately started on thiamine, D50 and folic acid supplementation for possible alcoholic encephalopathy. Soon, however, his diagnosis was refined to Cannabinoid-induced hyperemesis syndrome and the management plan focused on prevention of seizure activity. Owing to the infancy of research on CHS and lack of validated clinical guidelines, deficiencies in nutritional status were overlooked and thiamine or other vitamin levels were never assessed. The failure of clinical improvement in cognition then led to an extensive workup consisting of infectious panels, autoimmune studies, malignancy evaluations, and imaging. Lastly, having ruled out almost all etiologies and finding only bilateral thalamic hyperintensities on T2 FLAIR MRI, the patient was diagnosed with WE. This was validated when the patient started to improve once he was started on appropriate thiamine supplementation.

It is highly probable that early and continuous thiamine supplementation would have significantly reduced the extent of neurological deficits in this otherwise healthy high-functioning individual. Thiamine is a very low-cost intervention and is associated with almost no side-effects except mild local irritation when delivered intravenously [8].

## Conclusions

We hope that our case reminds providers to remain vigilant in identifying and treating all vitamin deficiencies early even in non-alcoholic patients. In addition, clinicians should foresee that increased cannabis use in our communities will also uncover other side effects which are currently less frequent.
